# Mechanistic experimental pain assessment in computer users with and without chronic musculoskeletal pain

**DOI:** 10.1186/1471-2474-15-412

**Published:** 2014-12-06

**Authors:** Hong-You Ge, Steffen Vangsgaard, Øyvind Omland, Pascal Madeleine, Lars Arendt-Nielsen

**Affiliations:** Center for Sensory-Motor Interaction (SMI), Department of Health Science and Technology, Faculty of Medicine, Aalborg University, Fredrik Bajers Vej 7D, 9220 Aalborg, Denmark; Clinic of Occupational Medicine, Aalborg University Hospital, P.O. Box 561, 9100 Aalborg, Denmark

**Keywords:** Sensitization, Pain mechanisms, Computer work, Conditioned pain modulation, Work-related musculoskeletal disorders, Pressure pain threshold, Experimental pain

## Abstract

**Background:**

Musculoskeletal pain from the upper extremity and shoulder region is commonly reported by computer users. However, the functional status of central pain mechanisms, i.e., central sensitization and conditioned pain modulation (CPM), has not been investigated in this population. The aim was to evaluate sensitization and CPM in computer users with and without chronic musculoskeletal pain.

**Methods:**

Pressure pain threshold (PPT) mapping in the neck-shoulder (15 points) and the elbow (12 points) was assessed together with PPT measurement at mid-point in the tibialis anterior (TA) muscle among 47 computer users with chronic pain in the upper extremity and/or neck-shoulder pain (pain group) and 17 pain-free computer users (control group). Induced pain intensities and profiles over time were recorded using a 0-10 cm electronic visual analogue scale (VAS) in response to different levels of pressure stimuli on the forearm with a new technique of dynamic pressure algometry. The efficiency of CPM was assessed using cuff-induced pain as conditioning pain stimulus and PPT at TA as test stimulus.

**Results:**

The demographics, job seniority and number of working hours/week using a computer were similar between groups. The PPTs measured at all 15 points in the neck-shoulder region were not significantly different between groups. There were no significant differences between groups neither in PPTs nor pain intensity induced by dynamic pressure algometry. No significant difference in PPT was observed in TA between groups. During CPM, a significant increase in PPT at TA was observed in both groups (P < 0.05) without significant differences between groups. For the chronic pain group, higher clinical pain intensity, lower PPT values from the neck-shoulder and higher pain intensity evoked by the roller were all correlated with less efficient descending pain modulation (P < 0.05).

**Conclusions:**

This suggests that the excitability of the central pain system is normal in a large group of computer users with low pain intensity chronic upper extremity and/or neck-shoulder pain and that increased excitability of the pain system cannot explain the reported pain. However, computer users with higher pain intensity and lower PPTs were found to have decreased efficiency in descending pain modulation.

**Electronic supplementary material:**

The online version of this article (doi:10.1186/1471-2474-15-412) contains supplementary material, which is available to authorized users.

## Background

Recent epidemiological studies suggest an increased risk of acute or transient pain complaints among computer users although a causal relation between work load and pain is still uncertain [1]. In addition to the intensity of computer use, a poor body posture and ergonomic design of the workstation also contribute to the occurrence of work-related musculoskeletal disorders (WMSD) in the upper extremity [2, 3]. Indeed, the development of musculoskeletal pain among computer users is related to multiple factors. Individual, physical, psychosocial, and organizational factors are reported to play important roles in the development of WMSDs [4]. In our recent cross-sectional epidemiological study, pain intensities for the last seven days and three months showed a strong positive association with pain duration in the forearm, elbow, neck, and shoulder regions among computer users [5]. This suggests that sustained musculoskeletal pain or peripheral nociceptive inputs from deep tissues may play a role in the chronification of pain or recurrent pain episodes among computer users. However, the pathophysiological mechanisms that initiate and maintain or retrigger pain in computer users are not well understood. It has been shown that widespread hyperalgesia and dysfunctional endogenous pain inhibition have been identified as characteristics of many musculoskeletal pain disorders [6, 7]. It is our hypothesis that the sustained or repeated episodes of musculoskeletal pain among computer users may influence the pain modulatory mechanisms. However, it is not known if modulatory mechanisms are altered among computer users with ongoing pain.

Conditioned pain modulation (CPM) is used to test the efficiency of descending pain control by utilizing two simultaneously applied painful stimuli (the ‘pain inhibits pain’ paradigm) [8]. CPM can be used to address the complex balance between the descending inhibition and descending facilitation on nociceptive processing. An impairment of the descending pain control has implications along the entire neuroaxis and can cause widespread hyperalgesia. In recent years the role of descending pain control has been studied intensely as it may be an important factor for the transition from acute to chronic pain [9]. The efficiency of CPM is reduced in many different chronic pain conditions including chronic musculoskeletal pain where widespread hyperalgesia is detected [10]. To document the existence of widespread muscle hyperalgesia pressure pain threshold mapping (PPT mapping) has been used to describe mechanical pain sensitivity in large body areas covering a muscle or several muscles [11, 12]. Likewise, dynamic pressure algometry has recently been developed as a technique to assess muscle hyperalgesia where the dynamic aspects are included in the evaluation as a roller with a pre-defined pressure applied across or along a muscle structure [13]. Quantitative sensory testing paradigms as mentioned above can assess the functional status of the excitability of the pain system in computer users with ongoing musculoskeletal pain but has so far not been applied to this group.

Thus, the purposes of the current study were (i) to assess sensitization by PPT mapping and dynamic pressure algometry (moving pressure stimulus), and (ii) to evaluate the efficiency of CPM among computer users with and without pain.

## Methods

### Participants

**Figure 1:**
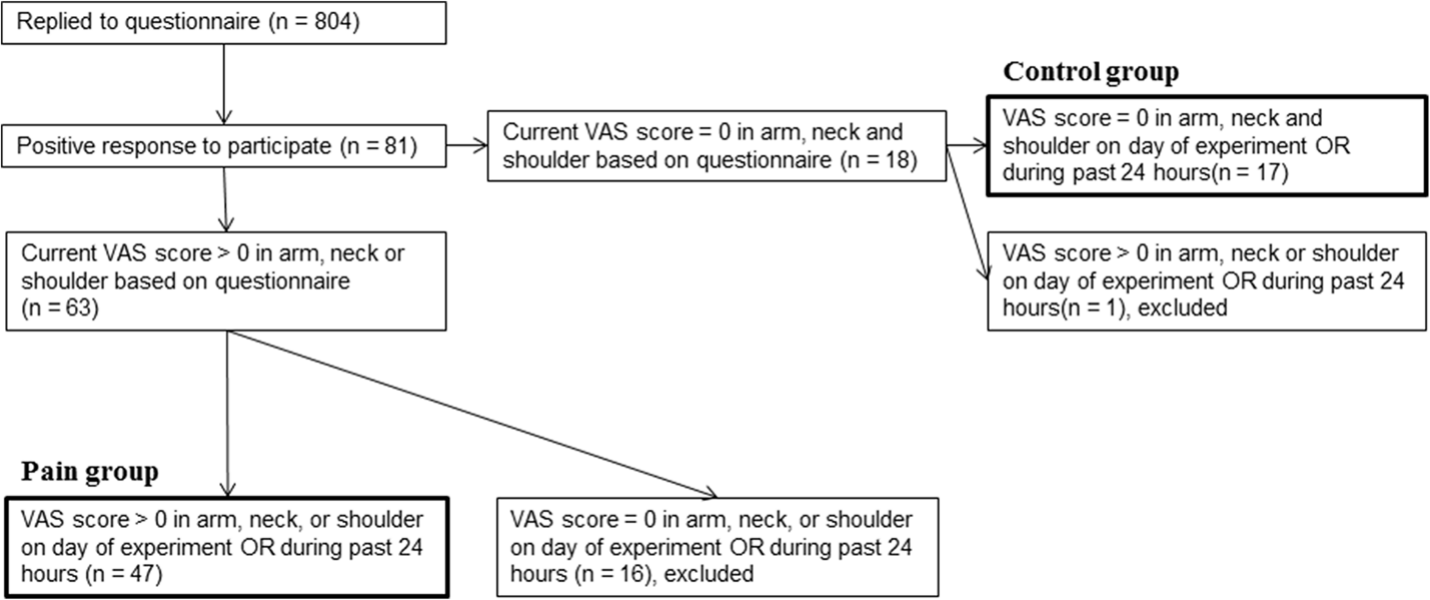
**Flow chart of the current study.** The sketch visualizes how the control group and the pain groups were selected. VAS: visual analogue scale of the pain intensity assessed on a 0–10 cm scale.

Sixty-four computer users (19 males and 45 females: mean age, 46.5 ± 1.2 years; mean BMI, 24.5 ± 0.5 kg/m^2^) with or without pain in the neck-shoulder and forearm regions were recruited to participate in the study. The study population was selected as a sub-sample of the 804 computer users who participated in a cross-sectional epidemiological investigation into the extent of computer use and the occurrence of WMSD [5]. The flow chart of the study is detailed in Figure 1. Participants reporting neck, shoulder, and/or arm pain intensity > 0 cm on the 0–10 cm visual analogue scale (VAS) in a structured web-based questionnaire [5] and during the past 24 hours or on the day of the experiment were assigned to the pain group. Participants reporting a pain intensity = 0 were assigned to the pain-free control group. Participants with pain due to other medical problems, such as disc prolapse, whiplash associated disorders, fibromyalgia or cervical nerve injury, were excluded. The study was approved by the local ethics committee of The North Denmark Region (No. N-20100048) and conducted in accordance with the Helsinki Declaration. Written informed consent was obtained from all participants prior to the experiment.

### Experimental protocol

The participants first rated their current pain intensity on the day of the experiment and the averaged pain intensity for the past 24 hours. The demographics and clinical characteristics of the participants were kept blinded to the experimenter responsible for the quantitative sensory testing. This one-session study consisted of three randomized sensory testing procedures: 1) PPT assessment in the neck-shoulder, in the elbow region and in the tibialis anterior muscle (TA), 2) dynamic pressure algometry in the elbow region, and 3) conditioned pain modulation (CPM) assessment. All recordings were made in the mentioned order for both groups.

### Pressure pain threshold mapping in the neck-shoulder region

Pressure pain threshold levels on the painful/most painful or dominant side of the neck-shoulder region were assessed using a pressure algometer at an application rate of 30 kPa/sec (Somedic, Hörby, Sweden). The PPT was defined as the minimum pressure first evoking a pain sensation. An upper cut-off limit of 1000 kPa was used. The algometer consisted of a 1 cm^2^ rubber tip plunger mounted on a force transducer. PPTs were measured twice (10 sec in between) for each point in random order, and the mean value was used for statistical analyses.

**Figure 2:**
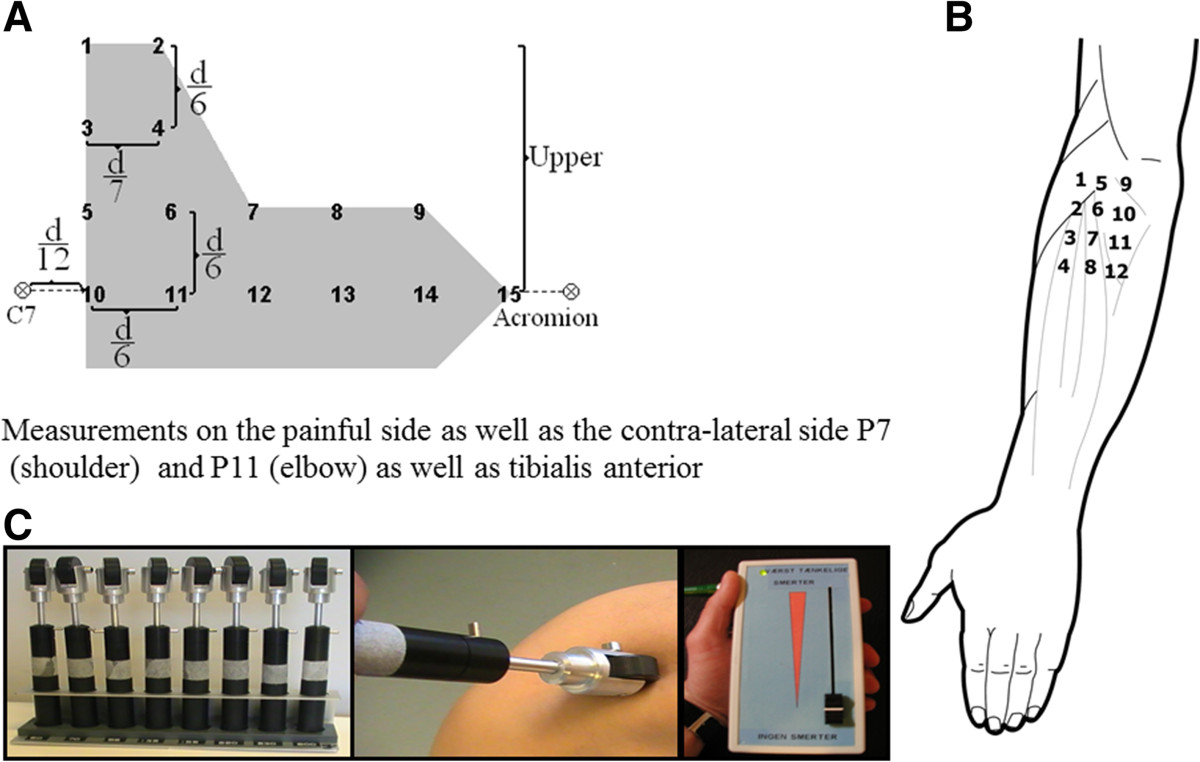
**The pressure pain assessment sites and the rollers used for assessing muscle tenderness.** Diagrams for pressure pain threshold (PPT) mapping in the neck-shoulder region **(A)** and in the elbow region **(B)** and the experimental set-up used for dynamic pressure algometry **(C)** showing a set of 8 rollers, the dynamic process, and the electronic visual analogue scale used to record pain ratings.

The mapping procedure in the current study followed a previous protocol [14]. To locate the assessment sites concisely, a wax pencil was used to mark a grid describing the locations from where the pressure stimuli should be applied. The grid for pressure point recording was set using the C7-acromion distance d (mean: 180 mm) to compute the inter-distance in a 15 point geometrically shaped grid covering the upper trapezius muscle (Figure 2A). Adjacent PPT points were separated by 1/6 of d (approx. 30 mm) except between point 1 and 2 and point 3 and 4 where the horizontal distance was 1/7 of d. In addition, PPT at point 7 on the contralateral side was also measured. Similar to the position for grid preparation, the participants were placed in a prone position during recordings. This provided a comfortable resting position for the participants and accessibility for PPT recordings. The recording order was randomized between points going either column or row-wise and by starting either at outer points and going inward or at inner points and going outward to prevent temporal summation [14].

### Pressure pain threshold mapping in the elbow region

The PPT levels on the painful/most painful or dominant side of the forearm extensors were assessed using the same algometer and same procedure as the PPT mapping in the neck-shoulder region. The mapping procedure also followed previous protocols [15, 16]. A wax pencil was used to mark the pressure point grid. PPT levels were assessed over 12 points forming a 3 × 4 matrix (4 points in the superior part, 4 points in the middle, and 4 points in the lower part around the lateral epicondyle) as follows: the lateral epicondyle was taken as the reference point (point 5). A line downwards from the lateral epicondyle was defined as the central column of the map. In this way, three vertical points separated by 20 mm were marked (labeled 6, 7 and 8). These four points were used for defining the remaining two columns of the matrix. The remaining points were symmetrically located 20 mm anterior (points 1 to 4) and 20 mm posterior (points 9 to 12) to each respective point (Figure 2B). Points 1 to 4 corresponded to the anatomical location of the musculo-tendinous junction (point 1) and muscle belly (points 2–4) of the extensor carpi radialis brevis, points 5–8 to the anatomical projection of the musculo-tendinous junction (point 6) and muscle belly (points 7, 8) of the extensor digitorum communis, and points 9–12 to the location of the musculo-tendinous junction (point 9) and muscle belly (points 10–12) of the extensor carpi ulnaris muscle as described previously [15, 16]. In addition, PPT at point 11 on the contralateral side was also recorded.

### Dynamic pressure algometry in the forearm

Dynamic pressure algometry is a newly developed method providing the opportunity to evaluate the pain reaction when rolling over a musculoskeletal structure with a given pressure which, as compared with static pressure pain threshold assessment, can elucidate other aspects of muscle hypersensitivity [13]. All the participants were placed in a supine position and the painful side of the forearm was exposed with the palm facing downwards. The painful or more painful side of the forearm was fixed with a positioning pillow (AB Germa, Kristianstad, Sweden). Three points were then marked at the wrist level: distal ends of the ulna and the radius, and the mid-point in between. At the elbow level, three points were marked: lateral edge of epicondyle, a point at the level of lateral epicondyle separating brachioradialis and extensor digitorum, and a mid-point in between. Connecting these points from the wrist to the elbow in parallel resulted in two separate tracks covering the finger extensor muscles. The dynamic pressure algometry set (Aalborg University, Aalborg, Denmark) consists of 8 rollers, each with a fixed load level of 500 g (approx. 50 kPa), 700 g (approx. 70 kPa), 850 g (approx. 85 kPa), 1350 g (approx. 135 kPa), 1550 g (approx. 155 kPa), 2200 g (approx. 220 kPa), 3850 g (approx. 385 kPa), and 5300 g (approx. 530 kPa) (Figure 2C). The experimenter maintained a constant pressure while the roller (contact area: 1 cm^2^, wheel diameter: 3 cm) was moving at a speed of 0.5 cm/sec on the predetermined tracks. While the roller (3.5 cm in diameter) was moving, the participants were asked to rate their pain intensity continuously on a 10-cm electronic VAS and the induced pain intensity was recorded.

In the current study, the PPT for the dynamic pressure algometry was determined at the mid-point of the extensor digitorum communis. Two rollers (different loads) were used. The first roller was chosen to correspond to the measured PPT. The second roller was selected at the load level just below the first roller. Thus, two different rollers were applied to the forearm on both sides on the more painful tracks starting from the wrist level and ending at the elbow level. The pain response was digitally recorded while the roller was moving along each track. The order of selection of tracks and rollers were randomized. The maximal pain intensity and the area under the VAS curve from the two sides for each roller were extracted and used for further analysis.

### Conditioned pain modulation

Tonic pain was induced in the non-painful or non-dominant arm by inflating a cuff (conditioning stimulation), and assessment of the pressure pain thresholds at the tibialis anterior (test stimulus) was performed before, during, and 5 min after the conditioning stimulation using handheld pressure algometry as previously described [17]. Briefly, a 7.5 cm wide tourniquet cuff (VBM, Germany) was wrapped around the non-painful arm. The lower rim of the tourniquet cuff was 3 cm proximal to the cubital fossa. The pressure was maintained just above the systolic pressure of each individual subject. After the target pressure was reached, each participant was asked to repeat a hand grip 10 times or more until a pain intensity of 4 cm was reached on the VAS. When 4 cm was reached on the VAS, PPTs at the tibialis anterior were assessed before, during, and 5 min after cuff-evoked pain subsided upon release of the cuff pressure. The PPT values obtained during cuff-evoked pain were normalized to baseline PPT values and used for further analysis.

### Statistical methods

Parametric and non-parametric statistical tests were used in agreement with normally and non-normally distributed data. The unpaired *t*-test was used to compare the differences between groups in PPTs at all measured points and to compare the differences between groups in pain ratings induced by dynamic pressure algometry. A two-way repeated measure analysis of variance (ANOVA) was used to compare the differences in PPT over time for CPM between groups. The Tukey test was used for post-hoc comparisons when appropriate. Spearman’s rank test was used for correlation analysis. The data are presented as mean ± standard error of the mean (SEM). The significance level was set to P < 0.05.

## Results

Table 1 reports the population characteristics. The job seniority was 19.3 ± 1.7 years for the pain group and 18.1 ± 2.4 for the control group (P = 0.71). The number of working hours/week using a computer were 38.4 ± 0.5 hours for the pain group and 36.9 ± 1.9 hours for the control group (P = 0.26). Further, the pain group reported pain in the neck/dominant shoulder (34 out of 47) and dominant elbow/forearm (36 out of 47).

**Table 1:** **Demographics and clinical characteristics of the computer users with pain and without pain**

	Pain group n = 47	Healthy controls n = 17
Male	14	5
Female	33	12
Age (yr.) (Mean ± SE)	47.6 ± 1.5	43.2 ± 2.3
Body mass index (kg/m^2^) (Mean ± SE)	24.1 ± 0.6	25.4 ± 0.9
Pain duration (yr.) (Mean ± SE)	6.8 ± 1.0	0
Pain intensity on the day of experiment (Mean ± SE)	2.3 ± 0.3 cm	0
Pain during last 24 h (Mean ± SE)	3.2 ± 1.8	0

### Pressure pain threshold mapping in the neck-shoulder region

**Figure 3:**
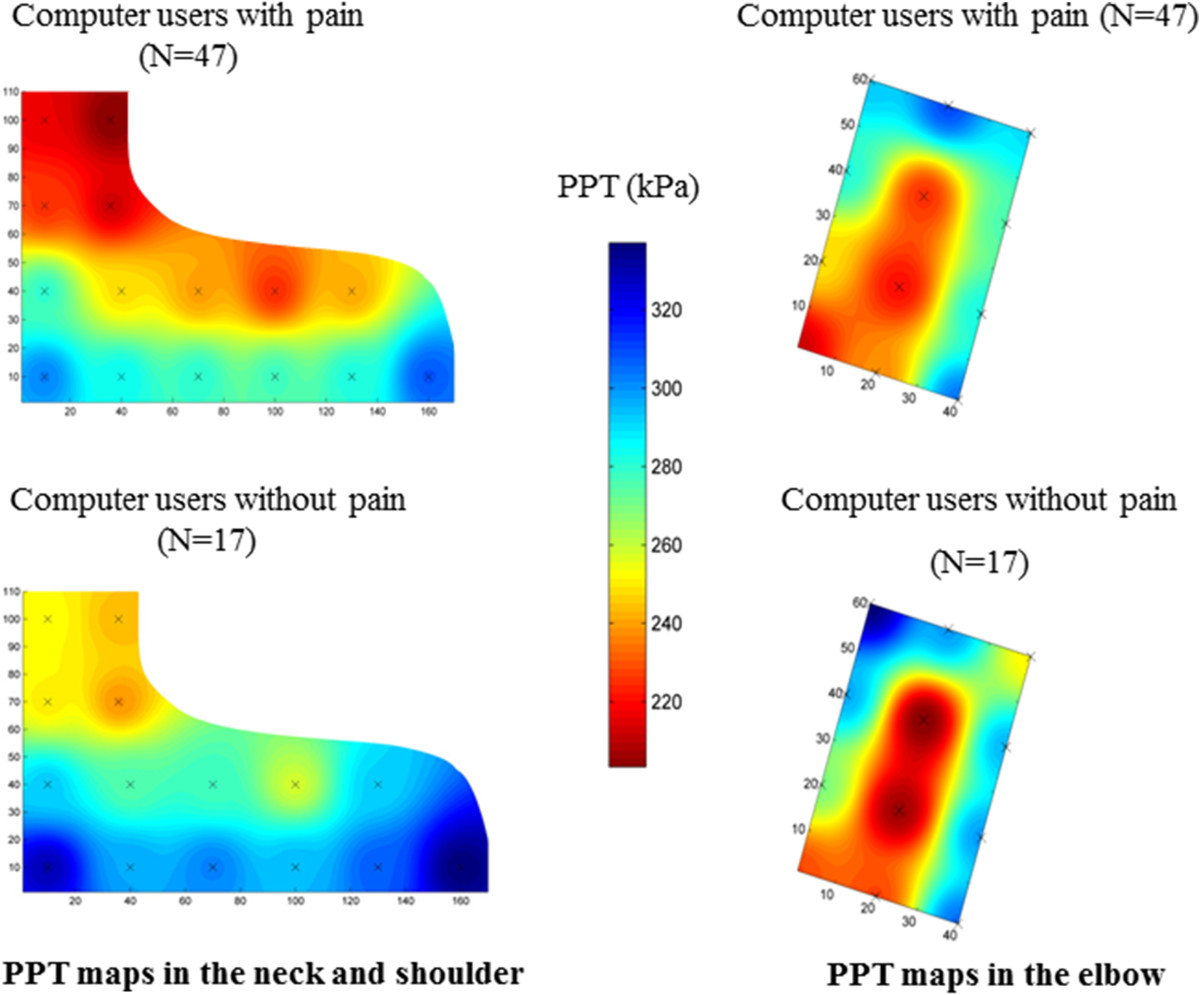
**Pressure pain sensitivity maps.** Pressure pain threshold (PPT) maps in the upper trapezius muscle show a trend of relatively lower PPTs among computer users with pain compared with pain-free controls (panel on the left). However, there was no significant difference in PPTs between groups (see also Table 2). PPT maps in the elbow show similar PPT levels between groups (panel on the right).

PPT mapping indicated a trend of pressure hyperalgesia in the neck-shoulder region in the pain group as compared with the control group (Figure 3, panel on the left). However, the PPTs measured at all 15 points were not significantly lower in the pain group than in the control group as shown in Table 2. Moreover, PPT at the neck-shoulder corner on the contralateral side (non-painful side) in the pain group was not significantly different from the painful side and not significantly lower than in the control group. The points with a relatively lower PPT in the pain group were located over the muscle belly and not over the musculo-tendinous junction of the upper trapezius muscle.

**Table 2:** **Pressure pain threshold (PPT) measured in neck-shoulder region among computer users with and without pain**

Point number	PPTs pain group (n = 47) kPa, Mean ± SE	PPTs healthy controls (n = 17) kPa, Mean ± SE	P value
1	216.1 ± 15.2	252.7 ± 28.2	0.23
2	203.4 ± 15.2	243.4 ± 25.1	0.17
3	225.8 ± 14.0	250.2 ± 29.5	0.41
4	212.8 ± 13.9	238.7 ± 27.9	0.37
5	279.7 ± 20.2	292.6 ± 35.0	0.75
6	248.6 ± 16.6	268.5 ± 25.9	0.42
7	240.2 ± 16.4	278.0 ± 26.8	0.24
8	225.0 ± 14.6	258.8 ± 40.4	0.33
9	241.3 ± 17.2	291.7 ± 50.1	0.23
10	302.0 ± 20.7	329.7 ± 29.2	0.48
11	285.3 ± 18.8	297.5 ± 29.9	0.74
12	280.6 ± 19.2	302.6 ± 32.6	0.56
13	277.9 ± 19.3	295.2 ± 47.2	0.69
14	282.1 ± 18.5	307.8 ± 48.4	0.54
15	308.6 ± 23.8	337.1 ± 40.3	0.54
7 on the contralateral side	234.4 ± 20.6	245.5 ± 26.9	0.27

### Pressure pain threshold mapping in the elbow region

There were no statistically significant differences in PPT between groups (Table 3). Further, there was no difference in PPT at point 11 between the painful and non-painful side in the elbow region (Table 3). The PPT mapping showed that the points with relatively low PPTs were populated over the muscle bellies, but not over the tendons, of the extensor carpi radialis brevis and the extensor digitorum communis (Figure 3, panel on the right).

**Table 3:** **Pressure pain threshold (PPT) measured in forearm region among computer users with and without pain**

Point number	PPTs pain group (n = 47) kPa, Mean ± SE	PPTs healthy controls (n = 17) kPa, Mean ± SE	P value
1	263.4 ± 16.8	285.9 ± 27.0	0.49
2	257.4 ± 16.7	265.1 ± 17.5	0.80
3	240.4 ± 14.8	251.1 ± 21.2	0.70
4	222.0 ± 15.9	230.3 ± 18.6	0.78
5	271.8 ± 18.4	266.1 ± 27.3	0.87
6	228.6 ± 15.3	217.3 ± 10.0	0.67
7	226.3 ± 15.6	219.2 ± 13.3	0.79
8	234.6 ± 16.0	230.1 ± 17.0	0.88
9	261.6 ± 19.5	242.1 ± 17.3	0.57
10	254.2 ± 15.4	264.5 ± 24.2	0.73
11	258.1 ± 16.5	266.1 ± 19.0	0.79
12	268.8 ± 18.7	269.3 ± 18.9	0.99
11 on the contralateral side	225.6 ± 19.5	265.4 ± 20.5	0.37
Mid-point in the tibialis anterior	418.9 ± 25.4	421.4 ± 40.2	0.87

### Pressure pain threshold in the tibialis anterior muscle

There was no significant difference in PPT at the TA muscle between the pain group as compared with the control group (Table 3).

### Dynamic pressure algometry in the forearm

**Figure 4:**
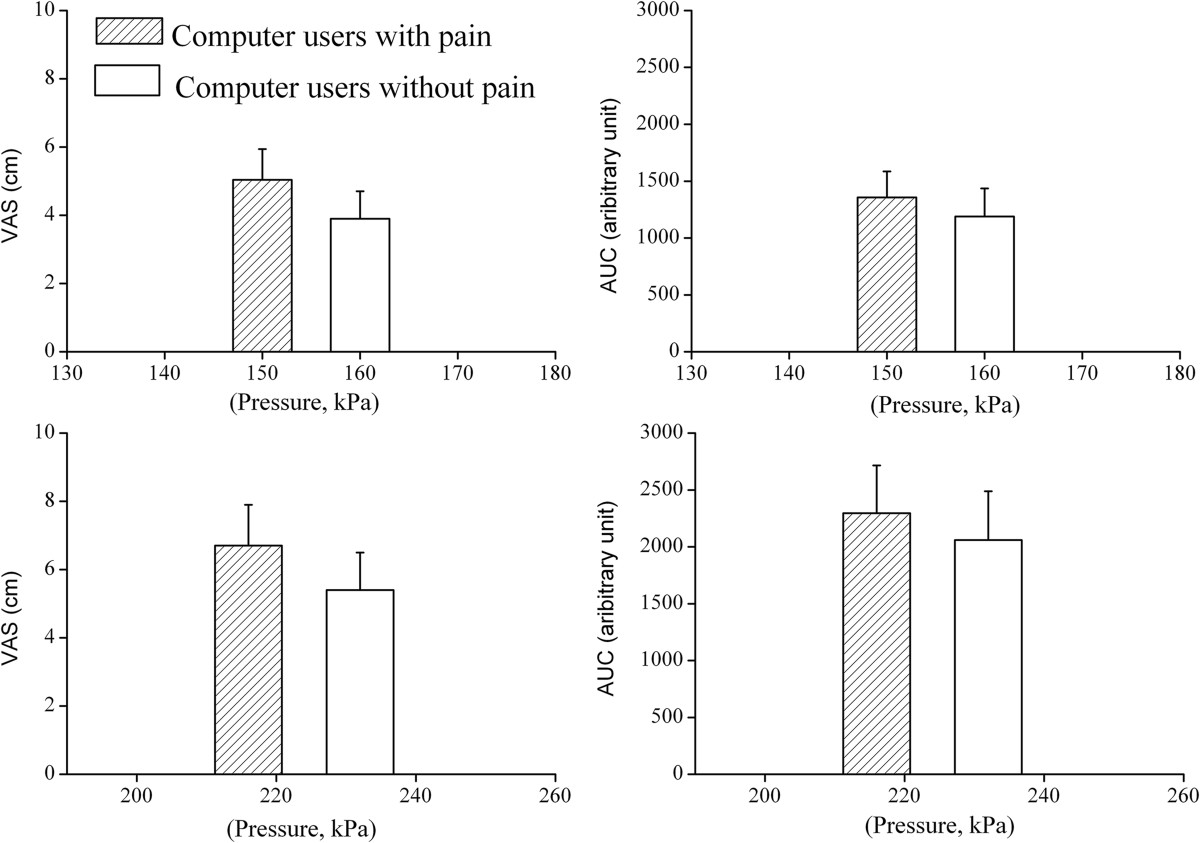
**Pain reactions to stimulation with the rollers.** Pain ratings during dynamic pressure algometry. Two graphs in the upper and the lower panels show the maximal pain intensity on the visual analogue scale (VAS) and area under VAS curve (AUC) induced by the roller with low pressure level and high pressure level, respectively. The roller with high pressure level corresponds to the pressure pain threshold (PPT); the roller with low pressure level corresponds to the pressure level just below PPT level (See methods section for further details). For both rollers, there was neither significant difference in maximal pain intensity nor in the AUC between groups. Pressure levels of the two rollers (mid-point on the x-axis in each column) were not significantly different between groups.

There were no significant group differences in the maximal pain intensity (P = 0.33) and the area under VAS curve (VASauc, P = 0.99) induced by the two rollers. Similarly, the pressure loads of the two rollers were not significantly different between groups (P = 0.51, Figure 4).

### Conditioned pain modulation

**Figure 5:**
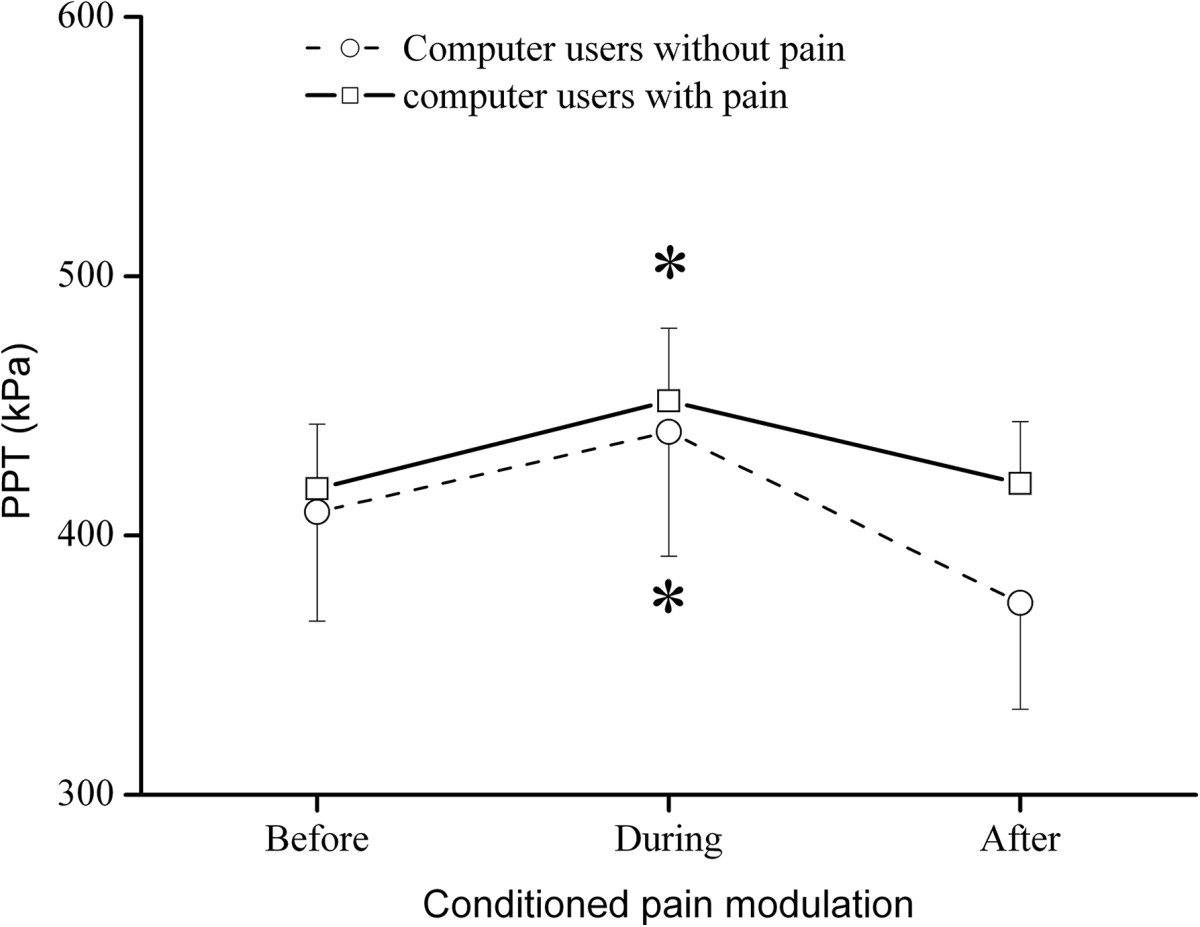
**The effect of conditioning pain modulation.** Conditioned pain modulation between groups. Significant changes (*: P < 0.05) in pressure pain threshold (PPT) were observed during conditioning stimulation as compared with before conditioning, but not between groups.

The two-way ANOVA revealed a significant difference in PPT over time (F = 9.25, P < 0.001), but without significant difference between groups. The post-hoc analysis revealed that PPTs during conditioning stimulation were significantly higher than before conditioning stimulation for both groups (P < 0.05, Figure 5) as an indicator of efficient CPM.

For the pain group significant correlations were found between normalized CPM assessed from tibialis anterior and (1) the pain intensity for the past 24 hours) (R = −0.29; P < 0.05) (more pain is associated with less efficient CPM), (2) mean PPT for the neck-shoulder region (pooled data from all 15 sites) (R = 0.32; P < 0.05) (lower PPT is associated with less efficient CPM), and (3) maximal pain intensity induced by the highest pressure level of rollers (R = −0.31; P < 0.05) (higher pain intensity evoked by the roller is associated with less efficient CPM).

## Discussion

The present study found no differences in response to quantitative sensory testing between computer users with and without low pain intensity chronic upper extremity and/or neck-shoulder pain. For the chronic pain group, higher clinical pain intensity, lower PPT values from the neck-shoulder, and higher pain intensity evoked by the roller were associated with less efficient descending pain modulation. This indicates a central involvement of the pain system in the group of computer users with stronger chronic pain intensities. An impairment of the descending modulation can be responsible for provoking widespread hyperalgesia.

### Sensitization among computer users

In the current study, PPTs were not significantly different bilaterally in the upper trapezius muscle in computer users with chronic pain as compared with pain-free computer users. Furthermore, the PPT levels and pain ratings evoked by the dynamic pressure algometer were similar indicating that the excitability of the pain system was also similar among computer users with and without ongoing pain.

Generalized hyperalgesia has been identified as a feature characteristic in many chronic musculoskeletal pain conditions, such as osteoarthritis [17, 18], fibromyalgia [19], chronic non-specific low back pain [20], and tension type headache [11]. Generalized hyperalgesia is assumed to represent augmented pain transmission at spinal and/or supra-spinal levels. The augmented pain transmission is usually associated with intense or sustained nociceptive stimulation [7]. In the current sample of computer users with musculoskeletal pain, the pain intensity was relatively low; with the mean pain intensity being around 2-3 cm on a 0-10 cm VAS. This choice was made in accordance with [5] as we considered that the participants of the control group should be pain-free computer users. Thus, the relatively low pain intensity from the peripheral tissues in the current study may not be sufficient to generate generalized hyperalgesia as compared to other chronic pain conditions with higher ongoing pain intensity (> 6 cm on a 0-10 cm VAS), such as fibromyalgia [12] and osteoarthritis [17, 18]. This is further supported by the fact that the generalized hyperalgesia is found to be correlated with the ongoing pain intensity [17]. The pain patterns of computer users are most commonly reported in the neck-shoulders, and least commonly for the hands, fingers, and/or wrists [5, 21]. Further, many of the computer users with WMSD report acute or transient pain complaints [1] which may not be sufficient to drive the generalized sensitization. The low mean pain intensity, high prevalence of pain focused in the neck-shoulder region, and transient pain characteristic may together account for the lack of differences in PPTs between groups in the neck-shoulder regions, in the forearm region, and in the TA muscle. This indicates that low pain intensity, transient pain (16 potentials participants from the pain group were excluded as they reported no pain), and a lack of spatial summation of pain from many different locations may not be able to induce generalized sensitization. Nevertheless, it is still unknown whether computer users with high pain intensity are associated with generalized hyperalgesia and as such this should be addressed in future studies.

In the pain group, PPT mapping of the upper trapezius muscle showed a trend of reduced PPTs in the upper trapezius muscle belly, but not in the tendon region, suggesting that some degrees of regional muscle tenderness may exist. Removal or decreasing peripheral nociceptive inputs from deep tissues has been shown to reverse central sensitization in fibromyalgia [19], knee osteoarthritis [22] and hip osteoarthritis [18]. Thus, future longitudinal and/or intervention studies may evaluate the role of peripheral nociceptive input and generalized sensitization among computer users with chronic musculoskeletal pain.

### Efficiency of conditioned pain modulation among computer users with musculoskeletal pain

The modulation of pressure pain sensitivity was observed in the current study in both groups. This suggests that the efficiency of CPM remains intact in computer users with a low level of chronic pain. In fact, reduced efficiency of CPM has been reported in many chronic pain conditions [23, 24]. The pain group in the present study showed a significantly negative correlation between ongoing pain and CPM efficacy further supporting the fact that the ongoing pain intensity is the primary driver for the potential impact on central pain processing. Previous studies also show that removal of peripheral nociceptive inputs results in normalization of CPM in osteoarthritis patients [22, 25]. Thus, the unaltered efficiency of CPM in the current study may be related to the relatively low pain intensity and the transient nature of pain reported by computer users [1]. Future studies may elucidate the relationship between the efficiency of CPM and the high level of chronic pain intensity in computer users.

The current study suggests that the efficiency of CPM may be decreased by a higher pain intensity of chronic pain. This is further supported by the fact that in the pain group of the present study a significantly negative correlation was found between the ongoing pain intensity and CPM efficiency. A higher ongoing pain intensity has been shown to be the primary driver for possible implications on the central pain processing in chronic osteoarthritis pain patients [17].

It is also noteworthy that increased muscle activity in the neck-shoulder and forearm regions has often been reported during computer work [26] and significant reduction of the pain intensity is achieved following ergonomic intervention programs and the improvement of workstations [2, 3]. Thus, pain from the muscle tissue may constitute a major peripheral pain generator in WMSD [27].

Therefore, successful management of regional pain in the neck-shoulder and upper extremities may eventually prevent pain chronification and avoid the development of dysfunctional central pain modulation and hence more widespread pain.

### Limitations of the study

This study has some limitations in terms of sample size. Further, the studied population reported a low level of pain as relatively common among computer users. However, the conclusion of the current study may not be generalized to the population of computer users with high chronic pain intensity. The pain assessments were made sequentially and this could lead to some carry-over effects. The participants rested for approx. 5 min between assessments. However, as no changes in, e.g., induced pain intensity and PPT, were observed, carry-over effects are not considered as a major confounding factor.

## Conclusions

The current study shows that computer users with a low level of chronic musculoskeletal pain were not associated with generalized sensitization and impairment of the descending pain modulation. Though in the pain group, the efficiency of CPM was significantly and negatively associated with clinical ongoing pain intensity, induced pain sensitivity in the elbow, and PPT values in the neck-shoulder region.

## Authors’ original submitted files for images

Below are the links to the authors’ original submitted files for images. Authors’ original file for figure 1 Authors’ original file for figure 2 Authors’ original file for figure 3 Authors’ original file for figure 4 Authors’ original file for figure 5

## Abbreviations

ANOVA: Analysis of variance
AUC: Area under VAS curve
CPM: Conditioned pain modulation
BMI: Body mass index
PPT: Pressure pain threshold
SEM: Standard error of the mean
TA: Tibialis anterior
VAS: Visual analogue scale
WMSDs: Work-related musculoskeletal disorders.
