# Supplementary figures and images for: Mechanistic experimental pain assessment in computer users with and without chronic musculoskeletal pain
Source: BMC Musculoskelet Disord. 2014 Dec 6;15:412. doi: 10.1186/1471-2474-15-412 (PMC4265505; doi:10.1186/1471-2474-15-412)

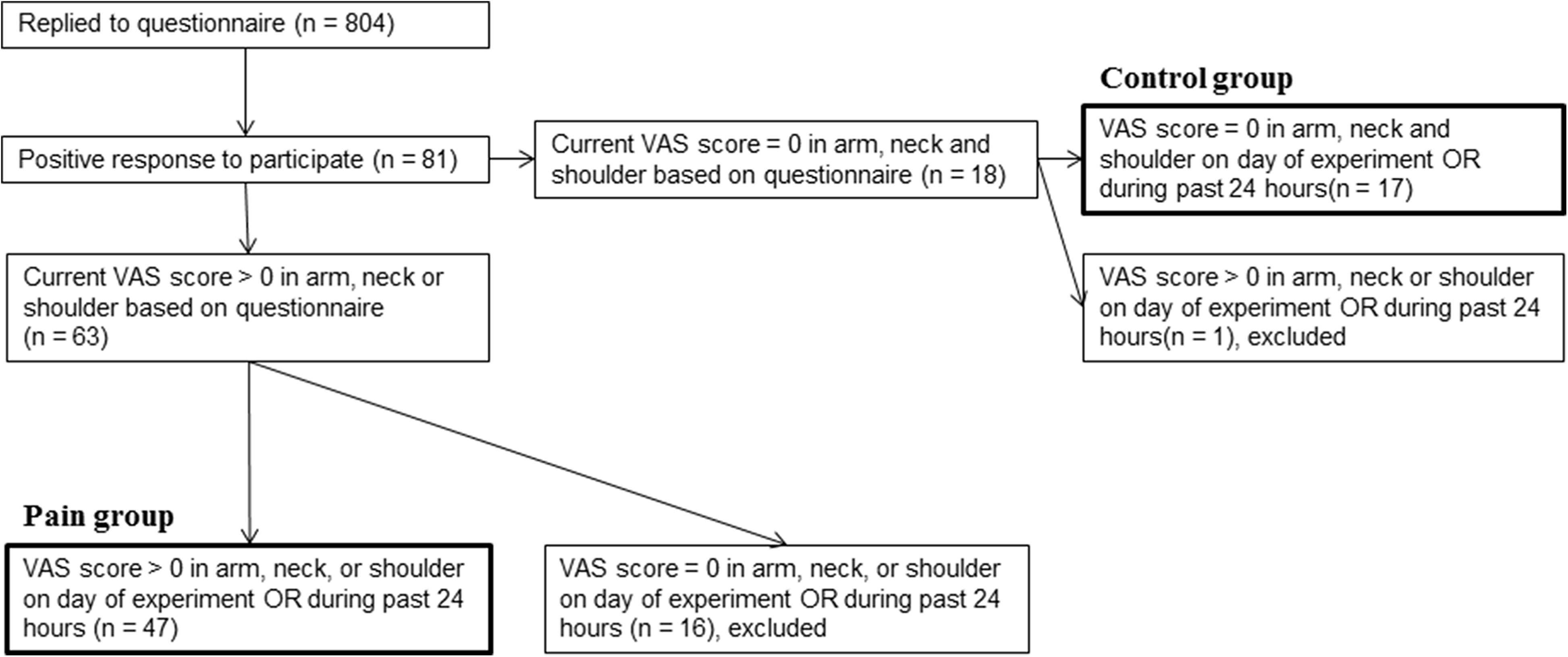

Supplement: Supplementary file 1 — Authors’ original file for figure 1 [file 12891_2014_2345_MOESM1_ESM.tif]

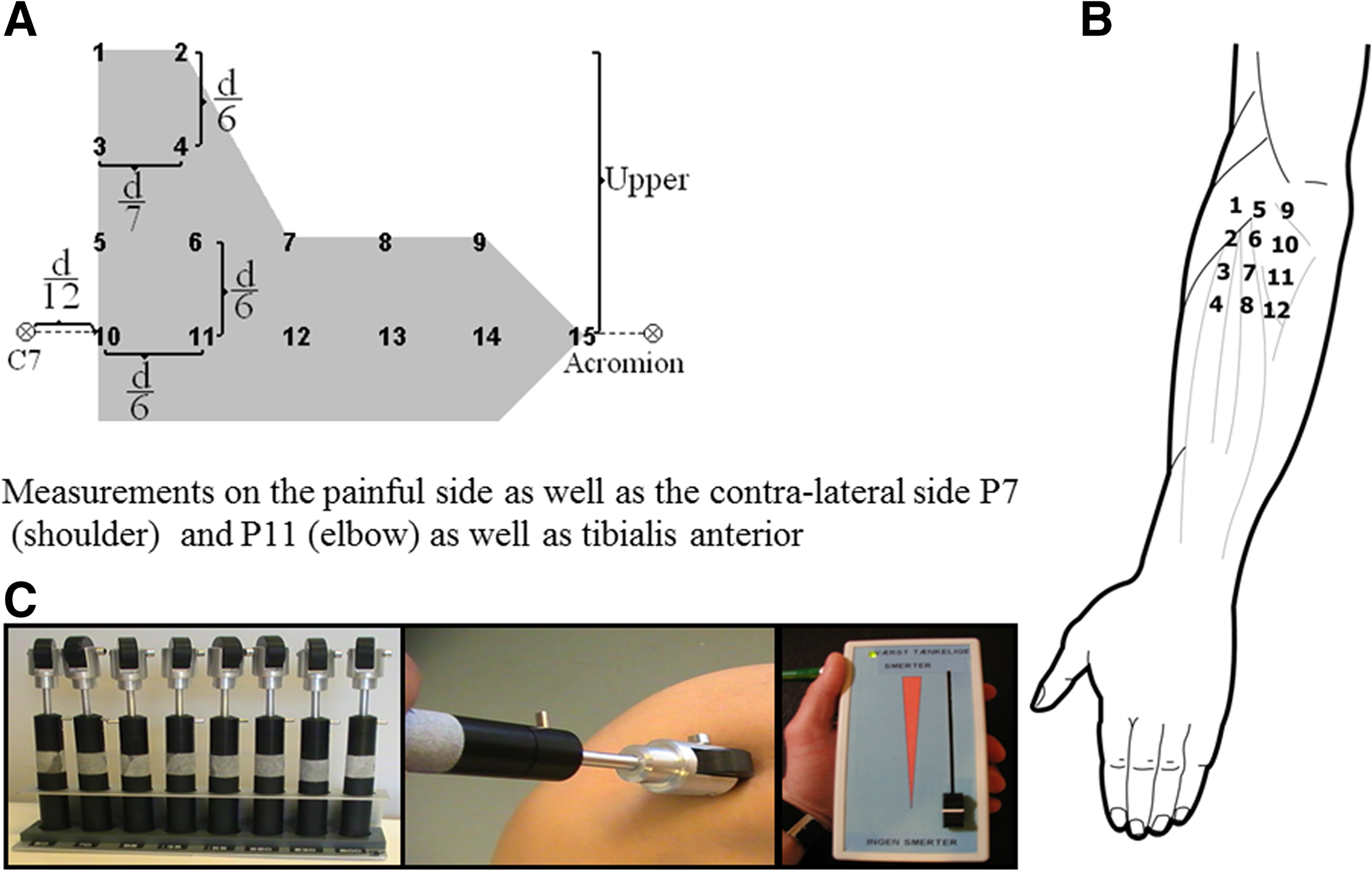

Supplement: Supplementary file 2 — Authors’ original file for figure 2 [file 12891_2014_2345_MOESM2_ESM.tif]

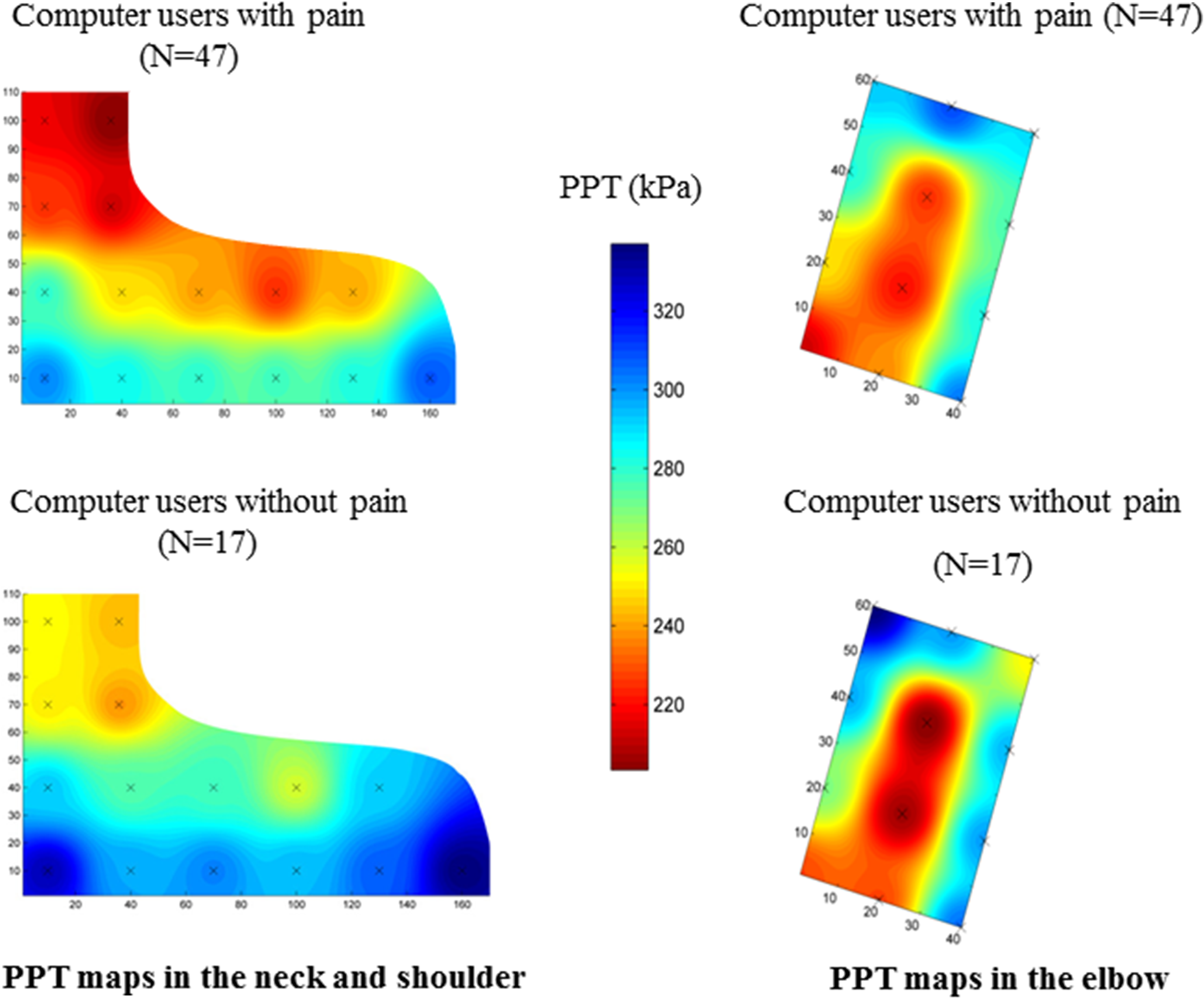

Supplement: Supplementary file 3 — Authors’ original file for figure 3 [file 12891_2014_2345_MOESM3_ESM.tif]

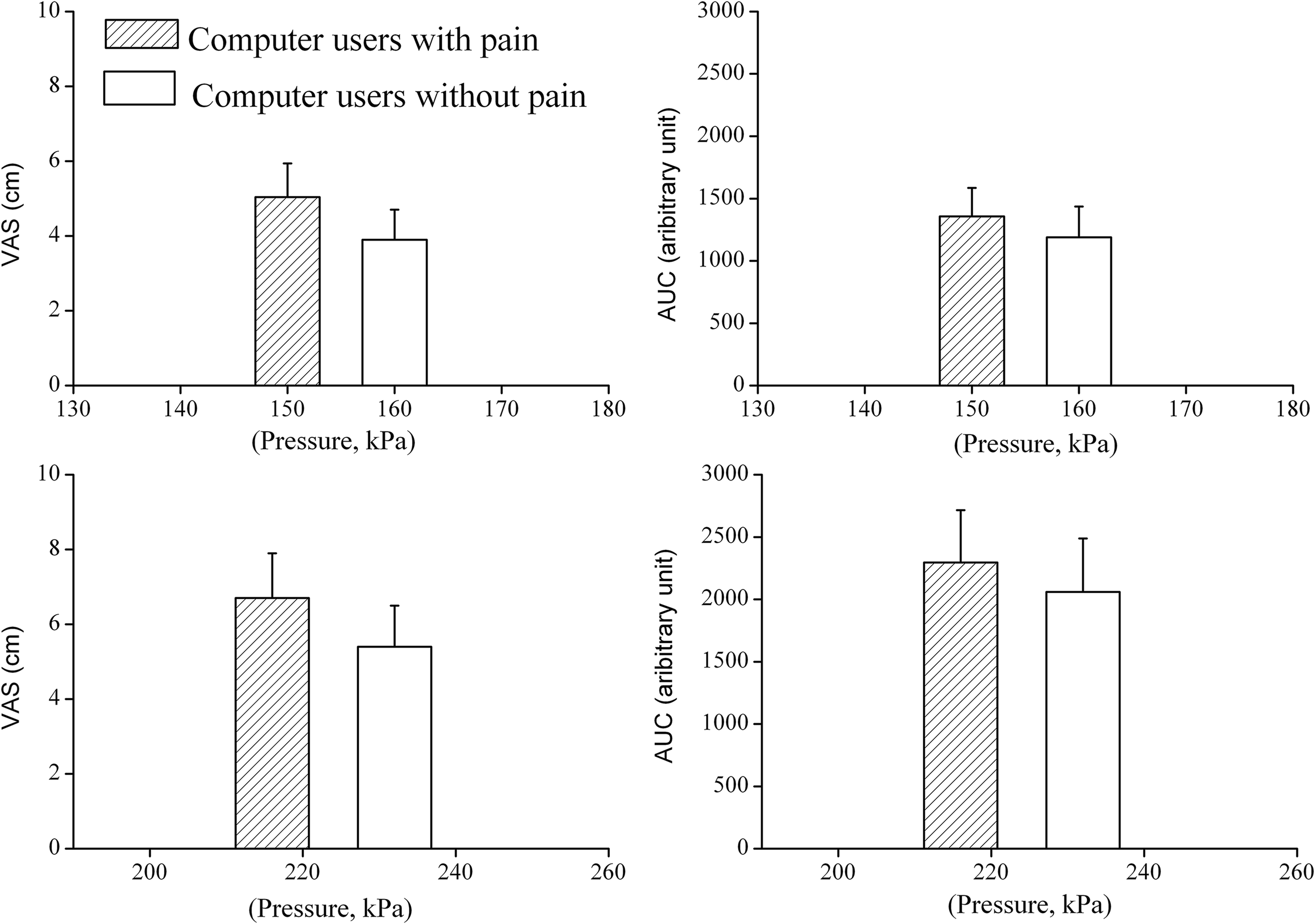

Supplement: Supplementary file 4 — Authors’ original file for figure 4 [file 12891_2014_2345_MOESM4_ESM.tif]

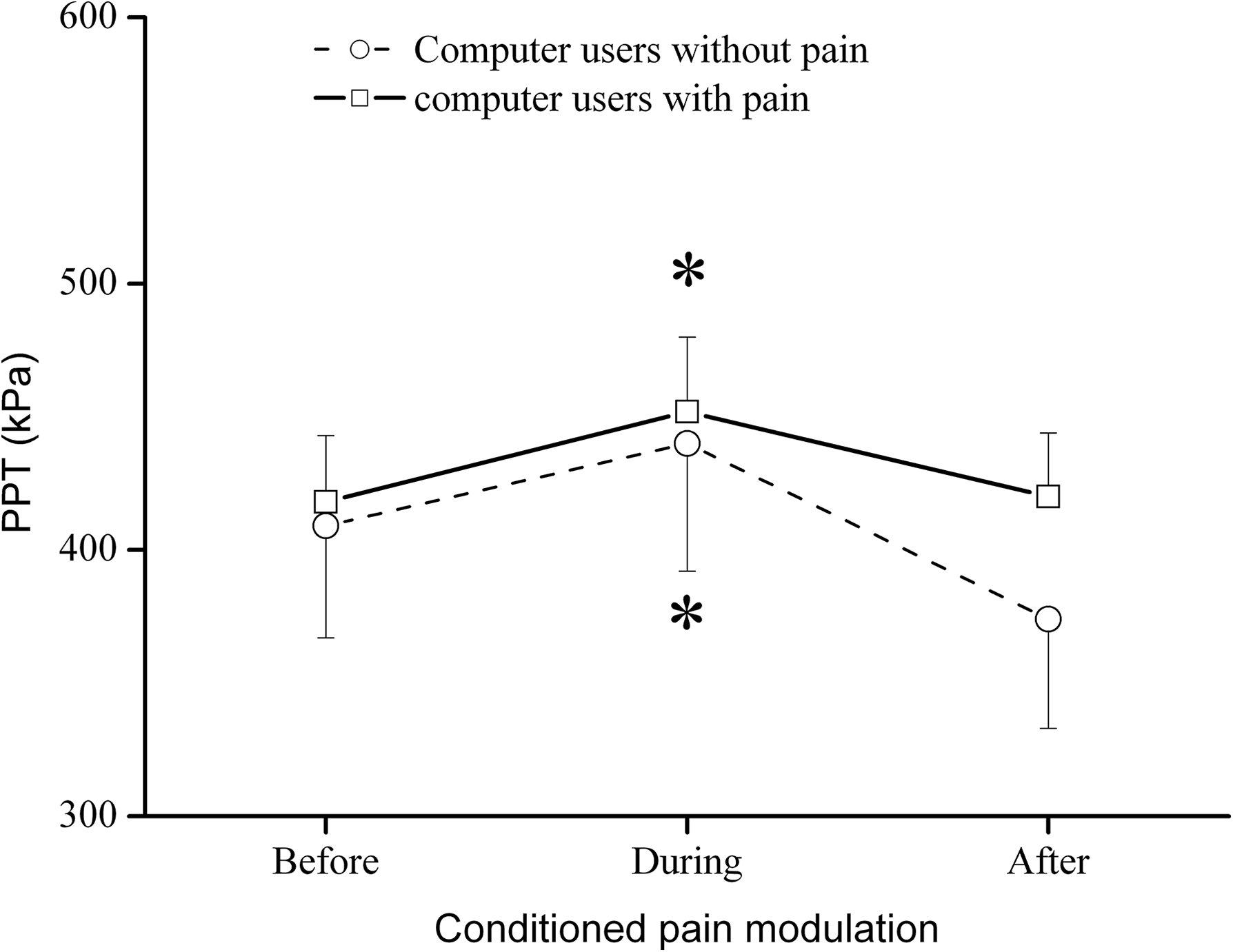

Supplement: Supplementary file 5 — Authors’ original file for figure 5 [file 12891_2014_2345_MOESM5_ESM.tif]
